# Perspective: Does Laboratory-Based Maximal Incremental Exercise Testing Elicit Maximum Physiological Responses in Highly-Trained Athletes with Cervical Spinal Cord Injury?

**DOI:** 10.3389/fphys.2015.00419

**Published:** 2016-01-14

**Authors:** Christopher R. West, Christof A. Leicht, Victoria L. Goosey-Tolfrey, Lee M. Romer

**Affiliations:** ^1^International Collaboration on Repair Discoveries, University of British ColumbiaVancouver, BC, Canada; ^2^School of Kinesiology, University of British ColumbiaVancouver, BC, Canada; ^3^Centre for Sports Medicine and Human Performance, Brunel University LondonLondon, UK; ^4^School of Sport, Exercise and Health Sciences, The Peter Harrison Centre for Disability Sport, Loughborough UniversityLoughborough, UK

**Keywords:** field tests, aerobic exercise, tetraplegia, cardiovascular system

## Abstract

The physiological assessment of highly-trained athletes is a cornerstone of many scientific support programs. In the present article, we provide original data followed by our perspective on the topic of laboratory-based incremental exercise testing in elite athletes with cervical spinal cord injury. We retrospectively reviewed our data on Great Britain Wheelchair Rugby athletes collected during the last two Paralympic cycles. We extracted and compared peak cardiometabolic (heart rate and blood lactate) responses between a standard laboratory-based incremental exercise test on a treadmill and two different maximal field tests (4 min and 40 min maximal push). In the nine athletes studied, both field tests elicited higher peak responses than the laboratory-based test. The present data imply that laboratory-based incremental protocols preclude the attainment of true peak cardiometabolic responses. This may be due to the different locomotor patterns required to sustain wheelchair propulsion during treadmill exercise or that maximal incremental treadmill protocols only require individuals to exercise at or near maximal exhaustion for a relatively short period of time. We acknowledge that both field- and laboratory-based testing have respective merits and pitfalls and suggest that the choice of test be dictated by the question at hand: if true peak responses are required then field-based testing is warranted, whereas laboratory-based testing may be more appropriate for obtaining cardiometabolic responses across a range of standardized exercise intensities.

## Introduction

With the advancement of the Paralympic movement over the last 10–20 years the physiological monitoring of Paralympic athletes, including maximal aerobic and anaerobic exercise testing in both the field and laboratory, is now common practice (Goosey-Tolfrey, 2010). Technological advances in treadmill and wheelchair roller design permit externally valid assessments of physiological parameters during wheelchair propulsion under carefully controlled laboratory conditions. The majority of studies that have assessed maximal exercise responses of elite athletes with cervical spinal cord injury (SCI) during wheelchair propulsion on a treadmill, including our own, have reported peak oxygen uptake values in the range of 0.8–1.6 L/min and maximal heart rate (HR) values in the range of 100–140 bpm, although the mean is typically around 120 bpm (Coutts et al., 1983; Wicks et al., 1983; Lasko-McCarthey and Davis, 1991; Schmid et al., 1998; Leicht et al., 2013; Paulson et al., 2013; West et al., 2014a). The dogmatic pathophysiological explanation for these relatively low values is purported to be loss of descending sympathetic cardiac control along with an attenuated catecholamine response and a decreased active muscle mass (Figoni, 1993; Hopman et al., 1998).

Recently, we reported that field-based exercise testing in elite wheelchair rugby athletes with cervical SCI elicits HR values of 140–180 bpm (West et al., 2014b). These values far exceed those collected in the same athletes during arm-crank ergometry and wheelchair propulsion on a treadmill (West et al., 2013). Further investigation revealed that a large number of these elite tetraplegic athletes (both rugby and hand-cycling) exhibit sparing of descending sympathetic fibers in the face of a motor and sensory compete injury (i.e., autonomic incomplete injury; Currie et al., 2015). Thus, it appears that factors other than disrupted descending sympathetic control may preclude the attainment of true peak physiological responses in the laboratory. To date, no study has specifically compared peak cardiometabolic responses between maximal field- and laboratory-based wheelchair exercise tests in highly-trained athletes with cervical SCI.

We have been collecting physiological data leading into the Beijing and London Paralympic cycles on the Great Britain wheelchair rugby squad. During this time, we have conducted a variety of field- and laboratory-based exercise tests on the same group of athletes, but have never directly compared peak physiological variables between laboratory- and field-based maximal wheelchair exercise tests. In the present study, we retrospectively reviewed our data and compared peak physiological responses between a standard incremental laboratory-based wheelchair treadmill test and two different field testing protocols.

## Materials and methods

### Data included

Nine male wheelchair rugby athletes with motor complete traumatic cervical SCI (C6-C7; 28.6 ± 2.6 year, 71 ± 16 kg, 1.80 ± 0.10 m, 7.1 ± 3.7 year post injury) were included into the study. The data were part of other research studies, some of which have been published elsewhere (8 of the present participants' 4 min push data, West et al., 2014b, and 8 of the present participants' maximal incremental test data Leicht et al., 2013; West et al., 2014a). All of the studies were approved by the University research ethics committee. In addition to peak physiological values, we extracted participant demographics and their International Wheelchair Rugby Federation (IWRF) classifications at the time of testing.

### Study design

Data were extracted for three different maximal exercise trials. Trial 1 consisted of a maximal 4 min field-based exercise test on a 110 m long indoor athletics track with a wide turnaround area at each end. Trial 2 (*n* = 7) consisted of a maximal 40 min field-based exercise test in a sports hall. Trial 3 consisted of an incremental wheelchair propulsion test on a treadmill. Athletes were thoroughly familiar with the testing protocols. Each trial was completed with athletes exercising in their own rugby wheelchair with regular strapping and gloves. Prior to each trial, athletes received the same standardized pre-test instructions, namely to void their bladder to minimize the chance of autonomic dysreflexia, and to avoid strenuous exercise for 24 h, caffeine for 4 h and food for 2 h prior to assessment. Trials 1 and 3 were performed between 1 and 8 months apart. Trial 3 was performed approximately 1 month after trial 2.

### Experimental trials

#### Trial 1

Athletes completed a maximal 4 min push on a 110 m synthetic indoor running track with minimal rolling resistance. Athletes pushed maximally in a straight line and were only required to turn at each end of the track where a wide area was provided to facilitate the maintenance of high speeds. Athletes were encouraged to cover as much distance as they could during 4 min. Environmental temperature ranged from 18.2 to 19.4°C, humidity from 40 to 42%, and barometric pressure from 737 to 739 mmHg.

#### Trial 2

Athletes completed a maximal 40 min push around a large sports hall. The push consisted of: a straight 40 m push along the first side, a 30 m zigzag push along the second side, a straight 40 m push along the third side and a 30 m backwards zigzag push along the final side. The athletes were encouraged to cover as much distance as possible during 40 min.

#### Trial 3

Athletes completed a maximal incremental wheelchair test to volitional exhaustion on a motorized treadmill with a moving rail to prevent falls (Saturn 300/125r, HP Cosmos, Nussdorf-Traunstein, Germany). Treadmill speed was kept constant and ranged from 2.0 to 2.8 m·s^−1^, depending on IWRF classification and previous performance during incremental treadmill exercise. The gradient was set at 1% and was increased gradually by 0.1–0.2% every 40 s. The maximal test was terminated when athletes were unable to maintain the treadmill speed, i.e., when they touched the spring of the safety rail for a third time. Standardized verbal encouragement was given throughout the test and push rate was freely chosen. All athletes underwent a standardized warm up as described elsewhere (Leicht et al., 2013). Environmental temperature ranged from 20.2 to 23.7°C, humidity from 27 to 61%, and barometric pressure from 741 to 758 mmHg.

### Methods of measurement

#### Heart rate

For trials 1 and 2, HR was measured beat-by-beat using a team system (Suunto team POD, Suunto Oy, Vantaa, Finland). For trial 3, HR was measured beat-by-beat using an individual HR transmitter coupled to a receiver (Polar Vantage NV, Polar Electro Oy, Kempele, Finland). HR_peak_ was defined for all trials as the highest HR averaged over a 5 s rolling window.

#### Metabolic

In trials 1 and 3, lactate concentration in haemolysed whole blood ([${\text{L}}_{\text{a}}^{-}$]_B_) was assessed at rest and immediately post-exercise using an automated analyser [Biosen C-line Sport, EKF Diagnostics, Barleben, Germany (Trial 1) or YSI 1500 SPORT, YSI Incorporated, Yellow Springs, OH, USA (Trial 2)]. In trial 3, oxygen uptake ($\dot{\text{V}}$o_2_) was assessed using an online system (MetaLyzer 3B, Cortex Biophysik GmbH, Leipzig, Germany). $\dot{\text{V}}$o_2peak_ was defined as the highest $\dot{\text{V}}$o_2_ over a 30 s rolling window.

### Statistics

Between-trial differences in physiological outcomes were assessed using either a one-way repeated-measures ANOVA (HR) or paired sample *t*-test ([${\text{L}}_{\text{a}}^{-}$]_B_). Relationships between peak physiological indices from field- and laboratory-based testing were assessed using Pearson's product moment correlation. Statistical analyses were carried out using STATA v12.0, with significance set at *p* < 0.05.

## Results

**Figure 1:**
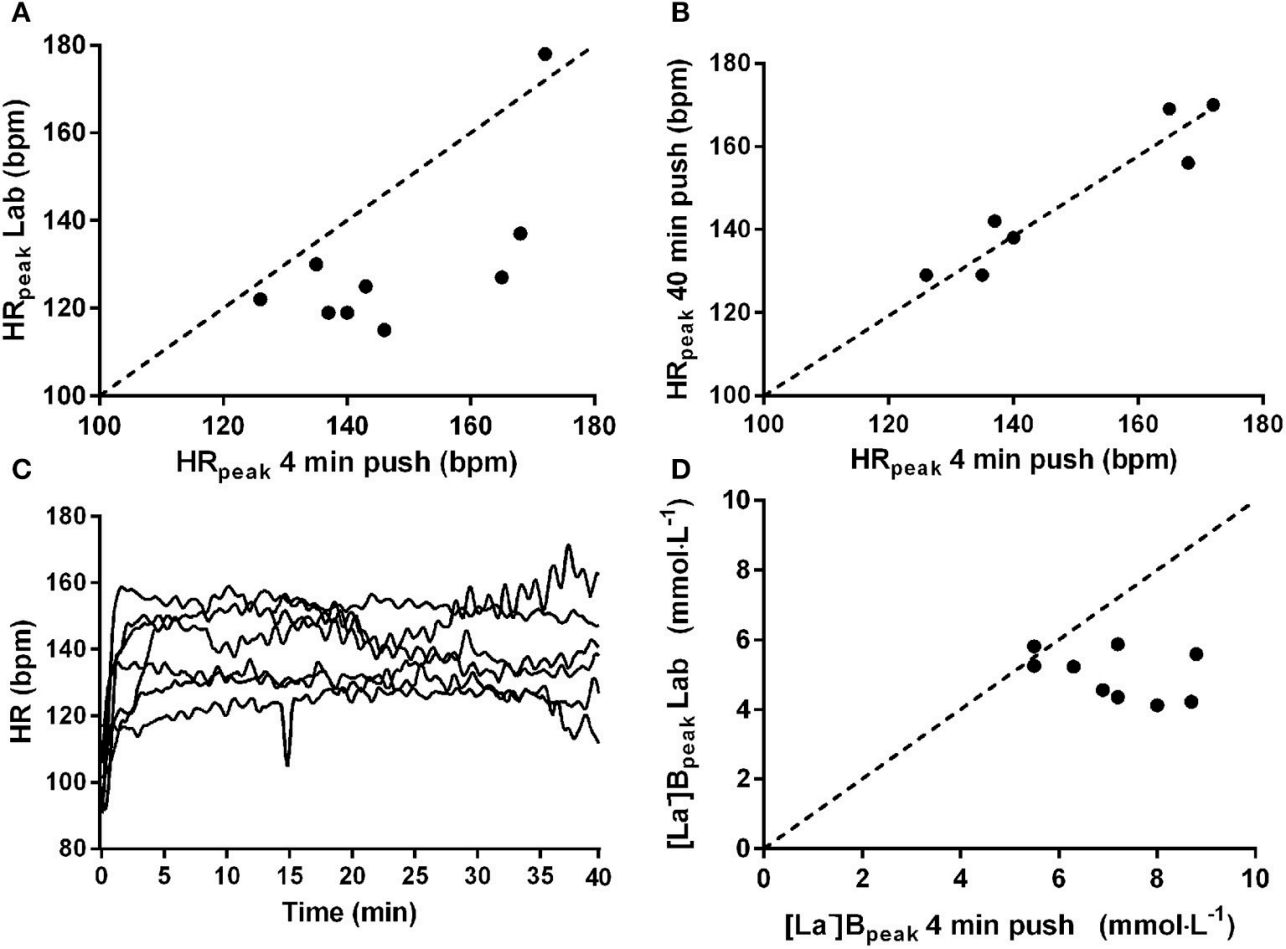
**Association between field- and laboratory-based peak heart rate (HR_peak_; A)**. Association between HR_peak_ during two different field-based assessments **(B)**. Individual HR responses to prolonged field-based exercise **(C)**. Associations between field- and laboratory based peak blood lactate concentration ([${\text{L}}_{\text{a}}^{-}$]_B_peak; **D**).

Individual athlete data for all trials are reported in Table 1. HR_peak_ was different between trials (*p* = 0.0035) and *post-hoc* testing revealed HR_peak_ was higher in trial 1 and trial 2 vs. trial 3 (*p* = 0.008 and *p* = 0.048, respectively). There was no difference in HR_peak_ between trial 1 and trial 2 (*p* = 0.29), and the values during both field-based exercise tests were strongly correlated (*r* = 0.88, *p* = 0.002; Figure 1B). There were no significant correlations between HR_peak_ achieved during the field-based tests and HR_peak_ achieved in the laboratory (*r* = 0.56–0.61, *p* > 0.08). During field-based testing, HR increased rapidly at the onset of exercise in all athletes and remained elevated throughout (Figure 1C). Blood lactate concentration was higher during trial 1 vs. trial 3 (*p* = 0.010; Figure 1D).

**Table 1:** **Individual peak physiological responses**.

			**Trial 1**		**Trial 2**	**Trial 3**				
	**Level**	**IWRF**	**HR (bpm)**	**[${\text{L}}_{\text{a}}^{-}$]_B_ (mmol/L)**	**HR (bpm)**	**Duration (min)**	**HR (bpm)**	**[${\text{L}}_{a}^{-}$]_*B*_ (mmol/L)**	**$\dot{\text{V}}$o_2_ (L/min)**	**$\dot{\text{V}}$o_2_ (ml/kg/min)**
1	C6	0.5	126	5.6	129	5.83	122	4.2	1.03	18.2
2	C7	1	146	5.5	Not collected	6.66	115	5.8	0.85	17.9
3	C6	1.5	142	6.9	Not collected	16.66	125	4.4	1.45	21.0
4	C7	2	169	5.3	157	13.33	137	5.2	1.47	23.6
5	C7	2.5	172	6.4	171	15.00	178	4.6	2.30	33.7
6	C7	2.5	135	7.2	139	9.41	130	5.9	1.42	21.8
7	C7	2.5	165	8.8	169	9.25	127	5.6	1.87	27.3
8	C7	2.5	148	5.5	150	7.86	119	5.3	1.98	27.3
9	C6	2.5	147	7.5	154	4.83	119	4.1	1.82	18.9
MEAN			150^*^	6.5^*^	153^*^	9.87	130	5.1	1.57	23.3
SD			16	1.2	15	4.19	19	0.7	0.46	5.3

*Trial 1: 4 min field-based maximal exercise test; trial 2: 40 min field-based maximal exercise test; trial 3: maximal laboratory-based incremental wheelchair propulsion test on a treadmill; IWRF, International Wheelchair Rugby Federation; HR, heart rate; [${\text{L}}_{a}^{-}$]_B,_ blood lactate concentration; $\dot{\text{V}}$o_2_, oxygen uptake*.

* *Significantly different from trial 3 (p < 0.05)*.

## Perspective

For the first time we report that peak heart rate and blood lactate concentration during maximal field-based exercise testing exceed values attained during maximal incremental laboratory-based wheelchair exercise on a treadmill. This suggests that incremental exercise testing in the laboratory, at least using the protocol described herein, does not elicit true peak cardiometabolic responses in highly-trained wheelchair rugby athletes with cervical SCI.

The HR values elicited during our laboratory-based treadmill test typify those reported in previous studies that have used wheelchair ergometry or treadmill exercise to investigate peak exercise responses in tetraplegic athletes (Coutts et al., 1983; Wicks et al., 1983; Lasko-McCarthey and Davis, 1991; Schmid et al., 1998; Paulson et al., 2013; Leicht et al., 2014; West et al., 2014a). An interesting observation from the two field-based trials compared to the laboratory trial was the push technique utilized. In the field-based trial, the athletes favored three small pushes followed by a short break for deep inhalation. During the push-phase many athletes also tended to “lean” into the abdominal strapping used to secure them into their sports chair. Anecdotally, athletes report that this push technique allows them to produce more force (power) with each push stroke. Leaning into the chest strapping likely compresses the abdomen and impairs diaphragmatic descent. In turn, this would be expected to reduce the force generating capacity of the diaphragm and may explain why athletes had to pause every three strokes for deep inhalation. On a treadmill, this technique is impossible to replicate as the wheelchair would roll to the back of the treadmill if pushing were to cease, thereby terminating the test. Thus, different push patterns may have been responsible for the lower cardiometabolic responses during treadmill exercise. To our knowledge, no study has directly compared maximal push mechanics between laboratory- and field-based testing in tetraplegic athletes. In able-bodied individuals, recent research suggests that current treadmill wheelchair propulsion protocols are unable to accurately reproduce the forces applied during field-based (i.e., over ground) propulsion (Mason et al., 2014). It is not yet clear whether these findings translate to highly-trained athletes with cervical SCI. An interesting observation was that the heart rate in three athletes (#1, 5, and 6) was similar between field- and laboratory-based testing. It is unclear why this was the case for these three athletes only. One explanation could be that these three athletes utilize a push technique that can easily be replicated in both the laboratory and field conditions. The idea of “transferability” of different propulsion techniques between laboratory and field settings has to our knowledge never been investigated in elite tetraplegic athletes but may provide important insight as to why some athletes can achieve similar maximal exercise responses in both the laboratory and field settings whilst others cannot.

Lower laboratory-based HR responses may also be a consequence of the inferior metabolic demand of incremental laboratory exercise compared to high-intensity constant load exercise. Increased acidosis associated with a higher blood lactate concentration in the field would be expected to drive greater peripheral and central chemoreceptor activation and augment central sympathetic outflow (Somers et al., 1989). In cervical SCI athletes with autonomic incomplete injuries, central sympathetic stimulation would elicit a direct and indirect (catecholaminergic) inotropic response. In autonomic complete athletes, it is possible that the sub-lesional sympathetic circuitry can still be activated from chemoreflexes via the pulmonary stretch receptors. Unfortunately, no studies have examined the interactions between chemoreceptor activation and vasomotor outflow after SCI. Moreover, while circulating catecholamines increase marginally during wheelchair ergometry in untrained cervical SCI (Schmid et al., 1998), no study has investigated the catecholaminergic response to field-based exercise. In our opinion, such studies are critical to advance our understanding of the physiological responses to exercise in athletes with cervical SCI.

The field-based measures of physiological performance reported herein are relatively crude, but are typical of those collected by researchers and/or sports physiologists during sports-specific field-based testing. We are yet to conduct field-based assessments of peak oxygen uptake using a portable metabolic cart. Such measures are the next step to confirm that peak cardiometabolic responses during laboratory-based exercise testing are indeed inferior to those obtained in response to field-based exercise testing. Nevertheless, we measured HR values that were considerably higher during both short- and long-duration field-based exercise compared to laboratory testing. Thus, future research should investigate why field-based exercise testing provides superior cardiometabolic responses (at least for most athletes) and seek to optimize maximal treadmill testing protocols. Until such studies are carried out we suggest that sports physiologists working in applied settings continue to use both laboratory and field-based testing and that the choice of test should be dictated by the question at hand as well as the availability of resources. Field-based maximal exercise testing provides superior external validity, the ability to accommodate large groups, and the free choice of push mechanics. Conversely, a laboratory-based exercise test allows for a more detailed physiological assessment under carefully controlled conditions with respect to protocol, temperature, and humidity.

## Considerations

We chose to use laboratory-based wheelchair propulsion to investigate peak responses because it is the most externally valid laboratory modality and because peak responses are slightly higher during wheelchair propulsion than during other laboratory modalities such as arm-crank exercise (Gass and Camp, 1984). Our decision to increment grade only was based on previous research that reported no significant differences in peak responses between treadmill protocols which increment speed, gradient, or a combination of both (Hartung et al., 1993). Finally, our participants were highly motivated wheelchair rugby athletes who were well versed in maximal incremental exercise testing. We are confident therefore that the laboratory testing environment was conducive to eliciting peak responses in the laboratory. That we measured similar HR_peak_ values during both field tests suggests that higher values in the field are indeed a real phenomenon and not an anomaly. Moreover, the mean values reported in the present study are almost identical to our previous field-based assessments of Paralympic hand-cyclists with cervical SCI (West et al., 2015). Finally, environmental conditions were similar between Trial 1 and 3 (not noted for Trial 2), suggesting differences in environmental conditions do not explain between-test differences in physiological responses. Thus, we are confident that the data presented herein represent true differences in physiological responses between laboratory- and field-based exercise testing.

## Concluding remarks

The present data imply that peak physiological indices measured in response to maximal incremental exercise testing in the laboratory using current protocols may not represent true maximal responses for athletes with tetraplegia. We suggest that future studies should investigate why field-based exercise testing provides superior cardiometabolic responses and seek to optimize maximal treadmill testing protocols to probe true peak responses in elite athletes with cervical SCI.

### Conflict of interest statement

The authors declare that the research was conducted in the absence of any commercial or financial relationships that could be construed as a potential conflict of interest.
